# Potential of patellar height measurement methods in predicting recurrent patellar dislocation incidence: a case-control study

**DOI:** 10.1186/s12891-023-06813-z

**Published:** 2023-09-05

**Authors:** Satoshi Yamashita, Shinya Ishizuka, Tadahiro Sakai, Hiroki Oba, Takefumi Sakaguchi, Takafumi Mizuno, Itaru Kawashima, Takashi Tsukahara, Shigeo Takahashi, Kazutoshi Kurokouchi, Shiro Imagama

**Affiliations:** 1 Department of Orthopedic Surgery, Nagoya University Graduate School of Medicine, 65 Tsurumaicho Showaku, Nagoya, 4668550 Aichi Japan; 2 Department of Orthopedic Surgery, Toyota Memorial Hospital, Aichi, Japan; 3 Department of Orthopaedic Surgery, Asahi University, Gifu, Japan; 4 Department of Orthopaedic Surgery and Arthroscopy Center, Juko Memorial Hospital, Nagoya, Japan

**Keywords:** Patellar height index, Patellar dislocation, Patellar alta, Patellar height measurement method

## Abstract

**Background:**

Recurrent patellar dislocation (RPD) is a multifactorial disease that affects young and active people. Patellar height measurements are used clinically to screen and diagnose knee conditions. However, there are no known studies that have assessed and compared the performance of patellar height indices for predicting the incidence of RPD, which could be used to recommend surgical treatment after primary patellar dislocation. This case-control study aimed to determine if the patellar height index could be used to predict the incidence of RPD, and to identify the optimal method in terms of its diagnostic ability for RPD.

**Method:**

Altogether, 133 patients (52 patients with RPD [Group R] and 81 sex- and age-matched controls [Group C]) were enrolled in this study. The Insall-Salvati (IS), Blackburne-Peel (BP), Caton-Deschamps (CD), and modified IS (mIS) methods were used to measure the patellar height index. The intra-observer and inter-observer reliabilities of these four methods were determined using intraclass correlation coefficients. A receiver operating characteristic curve analysis was performed to evaluate the predictive ability of each index and identify the cut-off values that indicated significantly increased risk of RPD.

**Results:**

Patient demographics were similar between the two groups. The inter-observer and intra-observer reliabilities were good for all four methods. In patients with RPD, the mean index values for the four methods were significantly higher than those in the matched controls. The area under the curve (AUC) values for IS, BP, CD, and mIS were 0.91 (standard error [SE], 0.03; 95% confidence interval [CI], 0.84–0.96), 0.72 (SE, 0.05; 95% CI, 0.63–0.81), 0.86 (SE, 0.03; 95% CI, 0.79–0.92), and 0.96 (SE, 0.01; 95% CI, 0.94–0.99), respectively.

**Conclusion:**

Patellar height indices had high predictive performance for the incidence of RPD. The mIS method had the highest AUC.

## Introduction

Recurrent patellar dislocation (RPD) is a multifactorial disease that commonly occurs in young and active people. [1, 2] Various anatomical abnormalities, such as lower limb mal-alignment, patella alta, trochlear dysplasia, and ligamentous laxity, are associated with patellar instability. Among these anatomical abnormalities, patella alta is reportedly one of the most important risk factors for RPD. [3–12].

Previous studies have demonstrated that the patellar height varies depending on the measurement method used. Although numerous different patellar height measurement methods have been described using lateral radiography or magnetic resonance imaging (MRI), [13] lateral radiography is more commonly used for outpatients with knee symptoms, because it is easy to perform and cost-effective.

Previous studies have investigated and compared the applicability, validity, and reliability of several patellar height measurement methods using lateral radiographs. Anagnostakos et al. reported the feasibility and applicability of five measurement methods (Blackburne-Peel [BP], Insall-Salvati [IS], Caton-Deschamps [CD], Linclau and Labelle-Laurin [LL]) during 90° knee flexion. [14] Seil et al. recommended the BP method because of its reliability and validity when determining patellar height. [15] Some indices of patellar height have been used to diagnose patella alta; [16] however, no previous studies have assessed and compared the performance of those indices for predicting the incidence of RPD.

Conservative treatment could be the first choice for primary patellar dislocation (PPD) without osteochondral fractures or other risk factors, including patellar alta, trochlear dysplasia, or lower limb valgus alignment, conservative treatment could be the first choice for primary patellar dislocation (PPD) [17, 18]. On the other hand, some recent studies reported that surgical treatment should be considered for patients with high risk of recurrence or highly active patients. [19–23] However, there is no clinically defined indications of surgical treatment for PPD. Suppose there were simple and easy methods to predict RPD incidence, it might be a valuable index to determine surgical treatment for the general population and sometimes for patients after PPD. Therefore, this case-control study aimed to determine if the patellar height index could be used to predict the incidence of RPD and highlighted optimal patellar height measurement methods that can be used in clinical practice. We hypothesised that one or some of the patellar height indices could have diagnostic ability to determine surgical treatment for the general population and sometimes for patients after PPD.

## Materials and methods

### Patients and ethical considerations

**Fig. 1:**
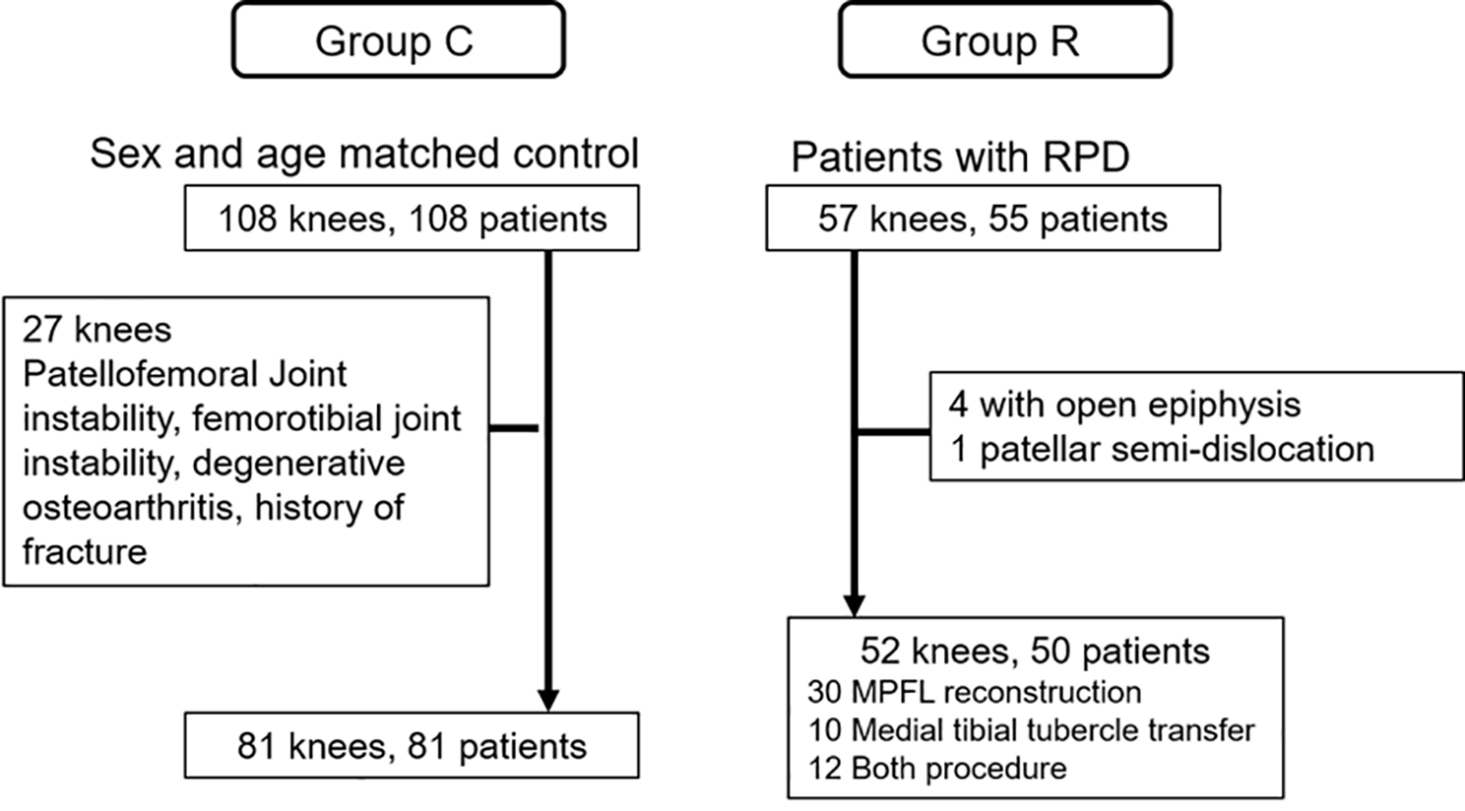
Flowchart of the study design. RPD, recurrent patellar dislocation; MPFL, medial patellofemoral ligament

This study was approved by the institutional review board of our hospital and complied with the Helsinki Declaration. Each patient provided written consent for study participation before enrolment. We investigated 57 knees of 55 patients with RPD who underwent surgery at our institution between January 2006 and April 2020. Exclusion criteria included permanent patellar dislocation, habitual patellar dislocation, nail patella syndrome, small patella syndrome, an open epiphysis of the distal femur or proximal tibia (diagnosed using the anterior-posterior view of knee radiograph) or a sub-dislocated patella at 30° of flexion (diagnosed using the axial view of knee radiographs in the supine position). RPD was diagnosed according to medical history as well as physical examination and radiographic findings. Five patients (four with an open epiphysis and one with a sub-dislocated patella) were excluded; therefore, 52 knees of 50 patients with RPD were assigned to Group R (Fig. 1). Among 52 patients, six patients dislocated just one time; however, they underwent surgery for recurrent sub-dislocation and strong apprehension. Twelve patients had dislocations twice, two patients had dislocations three times, ten patients had dislocations five or six times, and twenty-two patients had dislocations more than ten times and did not remember the accurate number of times they had dislocations.

Surgical procedures used in this study included medial patellofemoral ligament reconstruction (30 knees), medial tibial tubercle transfer (10 knees), or both procedures (12 knees). Subsequently, 108 patients who visited the emergency room after a traumatic event and underwent radiological examinations of their knee joints in our institution between January 2010 and December 2019 were enrolled as the control group (group C) and were matched for sex and age. They were diagnosed with knee bruise, strain, cut wound or excoriation at the emergency room or orthopaedic clinic. There was no patient with patellar dislocation in group C. Patients were excluded if they had chronic knee pain, fractures, patellofemoral joint instability, femorotibial joint instability, degenerative osteoarthritis, a history of lower limb fracture, previous knee injury, or surgery, and other knee disorders. After exclusion, 81 knees (81 patients) were assigned to group C. In total, 133 patients were enrolled in this study.

### Patellar height determination

**Fig. 2:**
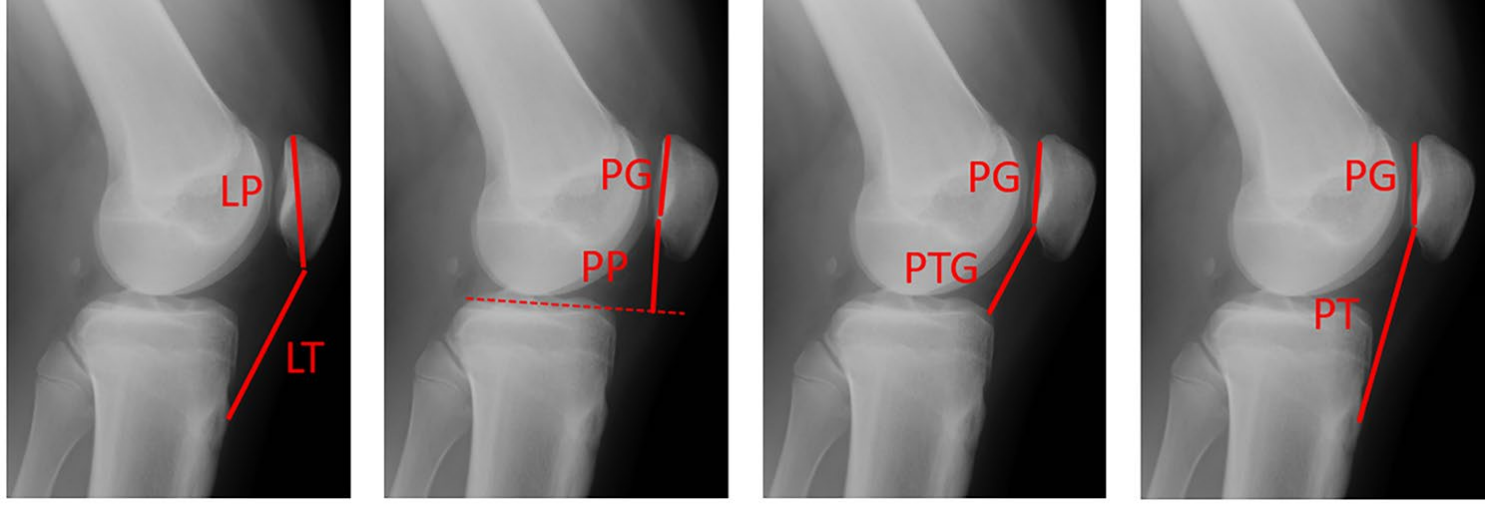
Details of the four patellar height measurement methods. The index is LT/LP for IS, PP/PG for BP, PTG/PG for CD, and PT/PG for mIS. IS, Insall-Salvati; BP, Blackburne-Peel; CD, Caton-Deschamps; mIS, modified Insall-Salvati; LT, length of the patellar tendon; LP, the longest portion of the patella; PP, shortest distance between the lower pole of the patellar articular cartilage and tibial plateau; PG, the length of the articular surface of the patella; PTG, the length between the inferior edge of the patellar joint surface and the antero-superior angle of the tibia

Patellar height was determined using lateral radiographs at a knee flexion angle ranging from 30° to 50° in both group C and R. The quality of the lateral knee was checked by two experienced orthopaedists before patella height measurement. All radiographs in group R were taken preoperatively. To measure patellar height, we chose four representative and commonly used methods: the IS, BP, CD, and modified IS (mIS) methods. All radiographs were evaluated by one experienced orthopaedist according to the methods shown in Fig. 2. LT indicates the length of the patellar tendon, LP is the longest portion of the patella, and PP indicates the shortest distance between the lower pole of the patellar articular cartilage and tibial plateau. PG indicates the length of the articular surface of the patella, and PTG is the length between the inferior edge of the patellar joint surface and the anterosuperior angle of the tibia. The index is LT/LP for IS, PP/PG for BP, PTG/PG for CD, and PT/PG for mIS.

### Statistical analyses

To determine intra-observer and inter-observer reliabilities, we calculated intraclass correlation coefficients (ICCs) with 95% confidence intervals (CI). To calculate inter-observer reliability, measurements were assessed at two different time points by two experienced orthopaedists who were blinded to patients’ data and conditions. Patella alta was diagnosed when the measured value was higher than 1.2 using IS, [24] 1.0 using BP, [25] 1.2 using CD, [26] and 2 using the mIS method [12], according to previously established criteria. Receiver operating characteristic (ROC) curve analysis was performed to evaluate the predictive ability of each index and identify a cut-off value that significantly increased the risk of RPD for each. The area under the curve (AUC) was calculated using the trapezoidal rule. AUC was defined as poor (0.5–0.7), acceptable (0.7–0.8), good (0.8–0.9), or excellent (> 0.9). Cut-off values were equivalent to the point at which the sensitivity and specificity were at their maximum. All data were analysed using SPSS ver. 26 (IBM SPSS Statistics 19.0; IBM). The two groups were compared using the Student’s t-test. Chi-squared analysis was used to compare groups with categorical variables. P-values < 0.05 were considered statistically significant. All variables were expressed as means and standard deviations with ranges.

## Results

**Fig. 3:**
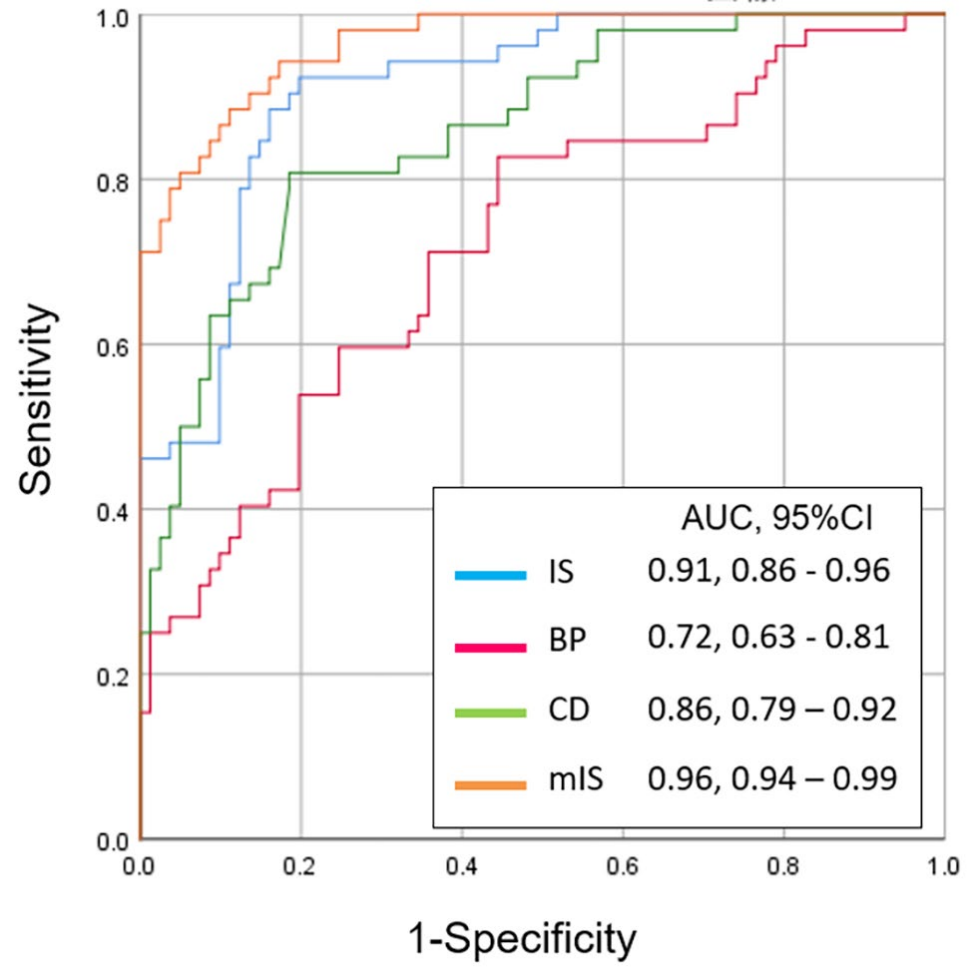
Receiver operating characteristic (ROC) curve for the IS, BP, CD, and mIS methods. IS, Insall-Salvati (blue line); BP, Blackburne-Peel (red line); CD, Caton-Deschamps (green line); mIS, modified Insall-Salvati (orange line); AUC, area under the curve; CI, confidence interval

Group C included 34 male and 47 female patients with an average age of 22.8 ± 7.3 years (range: 14–40 years). There were 32 right knees and 49 left knees. Group R included 20 male and 32 female patients with RPD with an average age of 20.5 ± 8.9 years (range: 12–52 years). There were 32 right knees and 20 left knees. The demographic characteristics were similar between the two groups (Table 1). The intra-observer and inter-observer reliabilities were good for all four methods (Table 2). In group C, 10 patients were diagnosed with patella alta using the IS method, 16 were diagnosed using the BP method, and none were diagnosed using the CD and mIS methods. In group R, 39 patients were diagnosed using the IS method, 24 were diagnosed using the BP method, 16 were diagnosed using the CD method, and 30 were diagnosed using the mIS method. The mean index values of the IS, CD, and mIS methods in patients with RPD were significantly higher than those in the matched controls (Table 3). The AUCs for the IS, BP, CD, and mIS methods were 0.91 (standard error [SE], 0.03; 95% CI: 0.86–0.96), 0.72 (SE, 0.05; 95% CI: 0.63–0.81), 0.86 (SE, 0.03; 95% CI: 0.79–0.92), and 0.96 (SE, 0.01; 95% CI: 0.94–0.99), respectively (Fig. 3). The cut-off value for each index to predict the incidence of RPD was 1.15, 0.91, 0.99, and 1.77 for the IS, BP, CD, and mIS methods, respectively (Table 4).

**Table 1:** Demographic data of Groups C (control) and R (recurrent patellar dislocation)

	Total	Group C	Group R	P-value
Number	133	81	52	
Mean age (years)	22.4	22.8	20.5	N.S.
Age range (years)	14–40	14–40	12–52	
Male/female	54/79	34/47	20/32	N.S.
Right/left	81/52	49/32	32/20	N.S.

N.S., no significance

**Table 2:** Intra-observer and inter-observer reliabilities of the four methods implemented in this study

Method	Intra-observer		Inter-observer	
	ICC	95% CI	ICC	95% CI
IS	0.924	0.859–0.966	0.912	0.795–0.964
BP	0.912	0.800–0.965	0.877	0.720–0.949
CD	0.915	0.801–0.965	0.901	0.768–0.959
mIS	0.963	0.910–0.985	0.934	0.845–0.973

IS, Insall-Salvati; BP, Blackburne-Peel; CD, Caton-Deschamps; mIS, modified Insall-Salvati; ICC, intraclass correlation coefficients; CI, confidence interval

**Table 3:** Mean index values of Groups C and R

		Group C (n = 81)	95% CI	Group R (n = 52)	95% CI	p-value^*^
IS						
	Index value	1.00	0.96–1.03	1.32	1.27–1.37	< 0.001
	Normal	71		13		
	Alta	10		39		
BP						
	Index value	0.89	0.86–0.93	1.05	0.99–1.1–	< 0.001
	Normal	65		28		
	Alta	16		24		
CD						
	Index value	0.9	0.87–0.94	1.14	1.09–1.20	< 0.001
	Normal	81		36		
	Alta	0		16		
mIS						
	Index value	1.58	1.54–1.62	2.08	2.01–2.16	< 0.001
	Normal	81		22		
	Alta	0		30		

IS, Insall-Salvati; BP, Blackburne-Peel; CD, Caton-Deschamps; mIS, modified Insall-Salvati; CI, confidence interval

**Table 4:** Cut-off values for the four indices

	IS	BP	CD	mIS
Cut-off value	1.15	0.91	0.99	1.77
Sensitivity	0.84	0.64	0.82	0.89
Specificity	0.86	0.73	0.83	0.86

IS, Insall-Salvati; BP, Blackburne-Peel; CD, Caton-Deschamps; mIS, modified Insall-Salvati

## Discussion

The present study compared four different methods of measuring patellar height and their ability to predict the incidence of RPD. Our findings revealed that the AUC associated with the mIS method was the largest (0.96). The AUC of mIS, 0.96 suggests excellent accuracy in predicting the incidence of RPD. It indicates that the patellar alta diagnosed using mIS methods could have the most reliable diagnostic ability which can differentiate patellar recurrent instability from asymptomatic stable patella. The pathogenesis of RPD is multifactorial, and previous studies have proposed many risk factors for RPD. Although the patella alta is recognised as an important predisposing factor for RPD, [4] there are few published studies focusing on the predictive performance of patellar height indices for the incidence of RPD.

Numerous studies have found that patella alta is associated with many patellofemoral pathologies, such as anterior knee pain, [1] patellofemoral cartilage lesions, [27] Osgood-Schlatter disease, [28] and patellar instability (including RPD). [15] [29] Larse et al. reported that the recurrence rate of patellar dislocation in patients with patella alta was 51%; the recurrence rate of acute patellar dislocation after conservative treatment ranged from 15–44%. [3] Several authors have compared the patellar height between patients with RPD and control subjects. Simmons et al. used the IS method and revealed that the mean index values were 1.02 and 1.58 in normal controls and RPD, respectively. [4] Furthermore, Dowd et al. used the IS method and reported that the mean index values were 1.03 in 50 knees of normal volunteers and 1.25 in 33 knees with patellar instability. [16] Our results for the control (1.00) and RPD (1.28) groups, according to the IS method, aligned with those of previous reports.

Several studies have compared several patellar height measurement methods in various knee conditions, including 90° flexion, [14] trochlea dysplasia, [29] in children, [30] and after valgus high tibial osteotomy. [31] Individually, these studies recommended one or two patellar height measurement methods, which varied based on the condition. However, most studies have compared the patellar height measurement methods in terms of their reliabilities and reproducibility. Aparicio et al. demonstrated that the CD method was more reliable and reproducible than the BP method in children. [30] Although the main purpose of our study was to evaluate the predictive performance for the incidence of RPD, we also analysed the ICCs. Our results indicated that the ICC scores for the four methods were good and similar or higher than the scores reported by previous studies. [32].

In this study, we used four representative measurement methods: the IS, BP, CD, and mIS. The IS and mIS methods showed the highest AUC values, which were 0.91 and 0.96, respectively. The IS method is one of the most studied and commonly used methods for the clinical diagnosis of patella alta. It is reported that both indices are independent of the tibial plateau; therefore, these methods mainly reflect the lengths of the patella and patellar tendon. [33] Although the mIS method has a similar concept to the IS method, the mIS index had a slightly higher AUC than the IS. While the IS ratio consists of patellar tendon and patellar lengths, the mIS ratio consists of the patellar tendon length and the articular surface length of the patella. Ward et al. reported that the patellar articular surface and its contact pressure are associated with patellar instability. [34] This could explain why the AUC value associated with mIS, which reflects the articular length of the patella, was slightly higher than that of the IS method. The BP and CD methods also had relatively high AUC values (0.72 and 0.86, respectively). These results indicate that while the BP and CD indices might be useful, they are less able to predict the incidence of RPD than the mIS and IS methods. The IS and mIS methods reflect the patellar tendon length, while the BP and CD methods reflect the patellar height when starting from the tibial plateau. Neyret et al. reported that the length of the patellar tendon is more specific and more sensitive than the CD index for predicting patellar instability. [35] Furthermore, Meyer et al. reported good long-term function of the patellar tendon following patellar tendon tenodesis for episodic patellar dislocation. [36] Our results may support these conclusions. Although the patella alta is an important factor for predicting patella instability, our results indicate that the patellar tendon length may be more important than the patellar position (starting from the tibial plateau). However, additional studies are needed to confirm these findings.

Previous studies have reported unsatisfactory results following conservative treatment for PPD. Stefancin et al. reported that conservative treatment resulted in a re-dislocation rate as high as 50%. [37] Furthermore, Thomas et al. reported that the rate of RPD was 45.1% after 10 years [5]. However, whether to perform surgical treatment for PPD is still controversial, and indications for surgery have not been established. Nwachukwu et al. suggested that patients with a high risk of RPD after PPD might benefit from surgical treatment. [21] Furthermore, several meta-analyses reported that surgical treatment reduced the rate of re-dislocation in PPD patients when compared to conservative treatments. [38–42] Hence, further studies are necessary to establish uniform criteria that clinicians can use when making decisions regarding surgery. Numerous studies have reported various risk factors associated with recurrent instability, such as patellar alta, trochlear dysplasia, small patella, general laxity, and valgus knee alignment. Recent studies have reported several classification systems that can be used to predict the re-dislocation after PPD. [17, 43, 44] However, there are no defined indications for surgical treatment for PPD when using the patellar height index. Currently reported classification systems could be accurate; however, they are also complex. Our study reports a high AUC value for mIS (0.96) and a recommended cut-off value (1.77). This value can be used to predict the incidence of RPD in the general population. Besides, this cut-off value helps to decide surgical indications for patients after PPD. The advantage of this method is its simplicity in measuring just patellar height without other predisposing factors, such as trochlea dysplasia, lower limb mal-alignment, and general laxity, to predict the subsequent incidence of RPD. Furthermore, a routine lateral knee radiograph might be needed for easy index measurement.

This study has some limitations. First, patellar height was determined using lateral radiographs and a knee flexion angle ranging from 30° to 50°. Therefore, small differences in the flexion angle might influence the index value recorded. Second, the number of patients is relatively small, and all the patients were Asian; therefore, the results may not be reflective of other races. Third, the control group in this study comprised patients without any patellar symptom. There is a possibility that patients without recurrence after PPD for a long time could be another better control group that could be used to identify the index for decision making during surgical treatment after PPD. Fourth, because this is the simple and easy method for predicting RPD incidence using just lateral knee radiograph, the other known risk factors including trochlea dysplasia, lower limb mal-alignment, and patient ages were not considered in this study’s analysis. Considering other associated risk factors, its diagnostic ability could be more helpful, and further studies seem to confirm this.

## Conclusion

The mIS method demonstrated the highest AUC; therefore, it could be preferred in predicting the incidence of RPD.

## Data Availability

To protect privacy and respect confidentiality; none of the raw data has been made available in any public repository. The original reports, laboratory studies, imaging studies and outpatient clinic records are retained as per normal procedure within the medical records of our institution, and available from the corresponding author on reasonable request.

## Abbreviation

RPD: Recurrent patellar dislocation
PPD: primary patellar dislocation
IS: Insall-Salvati
BP: Blackburne-Peel
CD: Caton-Deschamps
mIS: modified Insall-Salvati
